# Perceptions of students and faculty on NCAAA-accredited health informatics programs in Saudi Arabia: an evaluative study

**DOI:** 10.1186/s12909-024-05065-2

**Published:** 2024-03-15

**Authors:** Haitham Alzghaibi

**Affiliations:** 1 Department of Health Informatics, College of Applied Medical Sciences, Qassim University, Buraydah, Saudi Arabia; 2 Institute of Health Informatics, University College London, London, UK

**Keywords:** Health informatics, Bachelor’s in health informatics, Saudi universities, Education, Curriculums, Health information management

## Abstract

**Background:**

As the healthcare sector becomes increasingly reliant on technology, it is crucial for universities to offer bachelor’s degrees in health informatics (HI). HI professionals bridge the gap between IT and healthcare, ensuring that technology complements patient care and clinical workflows; they promote enhanced patient outcomes, support clinical research, and uphold data security and privacy standards. This study aims to evaluate accredited HI academic programs in Saudi Arabia.

**Methods:**

This study employed a quantitative, descriptive, cross-sectional design utilising a self-reported electronic questionnaire consisting of predetermined items and response alternatives. Probability-stratified random sampling was also performed.

**Result:**

The responses rates were 39% (*n =* 241) for students and 62% (*n =* 53) for faculty members. While the participants expressed different opinions regarding the eight variables being examined, the faculty members and students generally exhibited a strong level of consensus on many variables. A notable association was observed between facilities and various other characteristics, including student engagement, research activities, admission processes, and curriculum. Similarly, a notable correlation exists between student engagement and the curriculum in connection to research, attrition, the function of faculty members, and academic outcomes.

**Conclusion:**

While faculty members and students hold similar views about the institution and its offerings, certain areas of divergence highlight the distinct perspectives and priorities of each group. The perception disparity between students and faculty in areas such as admission, faculty roles, and internships sheds light on areas of improvement and alignment for universities.

**Supplementary Information:**

The online version contains supplementary material available at 10.1186/s12909-024-05065-2.

## Introduction

Health informatics (HI) is a multifaceted field dedicated to the collection, storage, retrieval, and utilisation of health data to enhance healthcare quality [1–3]. It combines methodologies from information science, computer science, and healthcare to improve healthcare delivery in various ways, such as electronic medical records, imaging, and decision support systems [2]. Offering bachelor's degree programs in HI is crucial for universities as the healthcare sector becomes increasingly reliant on technology [4–6]. HI professionals bridge the gap between IT and healthcare, ensuring that technology complements patient care and clinical workflows [7, 8]. These experts promote enhanced patient outcomes, support clinical research, and ensure that data security and privacy standards are met. With the growth of telemedicine and global health challenges, these professionals design and manage systems that cater to diverse populations and adapt to constantly evolving technological developments [4–6]. HI is an interdisciplinary field that combines elements of medicine, IT, management, and social sciences; an HI degree offers graduates a comprehensive understanding of modern healthcare challenges. As the healthcare industry continues its digital transformation, there is an indispensable need for a workforce trained in HI to ensure cost efficiency, address global health crises, and future-proof the healthcare sector for new technological developments [7]. However, despite the significance of this topic, no research has evaluated accredited HI programs in Saudi Arabia.

Increasing interest in the domain of human–computer interaction (HCI) and the need for individuals who are proficient in this discipline have led to a proliferation of educational prospects, including degrees at various levels [1, 5, 9]. The growing importance of the field led the International Medical Informatics Association (IMIA) to revise the framework of HI and medical informatics education in 2010 with the goal of addressing the educational requirements of a diverse group of healthcare professionals from many different fields, such as medicine, nursing, healthcare management, dentistry, pharmacy, public health, health record administration, and informatics. The HI competences framework was designed to fulfil the needs of specialized initiatives in the fields of biomedical research and HI [10]. HI education in Saudi Arabia has undergone a significant transformation as the country transitioned into a modernised healthcare system in the late twentieth century [11]. While the system was initially manual and paper-based, the country quickly recognised the need for digitised healthcare. Specialised HI programs were then established in universities such as King Saud University and King Faisal Specialist Hospital and Research Centre. The Saudi Association for Health Informatics (SAHI) was formed to further facilitate professional collaboration, and several educational institutions now offer comprehensive programs in the field. This highlights Saudi Arabia’s commitment to integrating technology into its healthcare system [12]. The bachelor of HI degree offered at Saudi universities are four-year programs consisting of courses in healthcare informatics, healthcare administration, and clinical informatics [6]. During their studies, students also have the opportunity to specialise in a particular field of HI that best represents their interests and helps them achieve their desired career goals. Students gain a strong understanding of information systems and HI as well as how to apply these concepts to healthcare. Finally, Saudi governmental universities are entirely supervised, monitored, and regulated by the Saudi Ministry of Education (MoE) [3, 13].

The Saudi National Commission for Academic Accreditation and Assessment (NCAAA) plays a pivotal role in ensuring the quality of higher education in Saudi Arabia, especially concerning bachelor’s degrees [14–16]. It establishes and maintains quality standards for academic institutions and their programs, offers accreditation to the programs which meet their standards, and continuously assesses institutions to promote ongoing improvement. The NCAAA also provides guidance, resources, and workshops to institutions, aiming to elevate the international recognition of Saudi academic credentials [16, 17]. Through its rigorous accreditation and assessment processes, NCAAA assures the public, students, and employers that the education offered by accredited institutions is high quality and relevant to current needs [14, 17]. By setting high standards, especially in research and innovation, the NCAAA supports Saudi Arabia's ambition of evolving into a knowledge-based economy and ensures that Saudi graduates remain competitive on the global stage [15, 16].

### Aim of the study

This study aims to evaluate bachelor’s degrees in HI programs accredited by the NCAAA in Saudi Arabia.

### Study objectives

This study measured the level of satisfaction of both students and faculty members, compared students’ and faculty members’ perceptions toward bachelor’s degrees in HI, and determined the level of quality of HI programs accredited by the NCAAA.

## Methods

This study employed a quantitative, descriptive, cross-sectional design, utilising a self-reported electronic questionnaire consisting of predetermined items and response alternatives [18–20]. To accomplish the objectives of this study, a self-designed questionnaire was created using Google Forms. The questionnaire used in this study was divided into three sections. The first section outlined the purpose of the study, emphasised the importance of participation, and informed the respondents of their ability to withdraw from the study at any time. Additionally, the section provided information regarding how the respondents’ information would be used and how their confidentiality would be maintained. The second section of the questionnaire gathered demographic data about the participants, including their gender, age, and the name of the university they attended. The third section of the questionnaire comprises eight primary categories. Each category reflects a crucial factor for assessing bachelor’s programs.

These factors were derived from previously published academic work regarding similar topics. Categories included facilities [21, 22], students’ involvements [23], curriculum [22, 24, 25], research [22, 26], admission [27–29], roles of faculty staff [22, 30–32], outcome [15, 33, 34], and internship [35–37]. For a comprehensive list of these aspects, please refer to Appendix 1. In addition, the factors were selected specifically to match NCAAA criteria [15, 38]. The participants were instructed to assess each item using a rating scale ranging from ‘1 = strongly disagree’ to ‘5 = strongly agree’. Despite the distribution of the two data-gathering instruments, these instruments exhibited a high degree of similarity. Students and faculty members completed the same questionnaires with the exception of the demographic information, which had been included to ensure that the survey was suitable for the specific populations being surveyed. Students were asked about their academic level while faculty members were surveyed regarding their professional status.

### Data collection instrument validity

The initial instrument underwent a comprehensive evaluation process by expert panels comprising external specialists, such as the academic department head, heads of student affairs and academic departments, and faculty members. The primary objective of the expert panel was to analyse the content of the questionnaire, determine its relevance to the intended population, and assess the clarity and comprehensibility of the questions. In response to the input received, the study incorporated demographic questions regarding the participants’ gender and age. Modifications were also made to the Likert-scale responses, reducing the number of response options from seven to five.

After receiving assessments and input from expert panels, a pilot study was conducted with a limited number of students (*n =* 9) and faculty members (*n =* 4). These participants were selected based on several factors, including diverse positions, gender, and study level. The objective of the pilot study was to obtain feedback regarding the quality of the questionnaire, including assessments about readability, comprehensiveness, appropriateness, and clarity. Participants were also asked to offer recommendations for enhancing the questionnaire.

Every individual involved in the pilot project successfully completed the initial version of the questionnaire and offered their observations and input on many aspects, such as the method, duration of questionnaire administration, and comprehensibility of the questions. The pilot study’s findings suggested that there was no need to include or exclude any questions. In general, the questionnaire was perceived as relatively clear and straightforward to administer. The user’s text was revised to enhance clarity and readability, with minor spelling, grammatical, and numbering corrections made based on the input. The pilot study was conducted during two weeks in January 2023. The input provided by the volunteers was integrated into the final iteration of the questionnaire as the necessary adjustments were modest.

### Data collection process

After both data collection instruments were modified and validated, Google Forms links were sent to the academic department heads at universities that met the inclusion criteria described below. The department heads were asked to share the links with their students and faculty members. The questionnaire was distributed during the week of February 06, 2023. Two reminder emails were sent to the departments heads during the week commencing 20 February 2023 and the week commencing 27 February 2023.

### Population and sampling

In this study, the targeted population consisted of all undergraduate students (*n =* 614) and faculty members (*n =* 85) enrolled in HI programs at Saudi universities that met the study’s inclusion criteria. Probability stratified random sampling were utilized [39, 40], so the inclusion criteria were Saudi universities offering HI programs at the bachelor’s degree level with internship programs. Male and female students and faculty members from all academic levels and positions were eligible to participate in the study. Those in medical informatics and biomedical informatics programs were excluded, as were postgraduate HI degree programs, bachelor’s degree in HI programs without internships, and programs that had not yet obtained NCAAA accreditation. A purposive, total population sampling method was used [39, 40]. Seven Saudi universities met these inclusion criteria. Using the selected sampling technique, questionnaires were distributed to 614 students and 85 faculty members at these universities.

### Data analysis

The questionnaire data were categorised into numerical groupings and subsequently input into IBM SPSS, Version 29. Cronbach's alpha was used to conduct an initial reliability test. Subsequently, an initial descriptive analysis was performed using the data obtained from the questionnaire. Furthermore, inferential statistics were used to ascertain any noteworthy disparities among the groups or associations between variables.

## Results

Cronbach’s alpha showed that the data collection instrument was statistically reliable (a = 0.86). The response rates were 39% (*n =* 241) for students and 62% (*n =* 53) for faculty members.

Table 1 presents a detailed breakdown of the faculty members and students based on various attributes. The majority of faculty members were men (58%, *n =* 31), but the distribution was more even in students. The highest percentage of students (69%, *n =* 166) were between 18 and 20, while the faculty had a more dispersed age range.

**Table 1:** Distribution of participants based on gender, age, faculty staff position, and students’ level of study

Faculty members			Students		
**Gender**					
	n	%		n	%
men	31	58	**Men**	124	51
Women	22	42	**Women**	117	49
Total	53	100	**Total**	241	100
**Age**					
	n	%		n	%
18–24	16	30	**18**–**20**	166	69
25–34	14	26	**21**–**25**	51	21
35–44	13	25	**26**–**30**	24	10
45–54	8	15	**Total**	241	100
55–64	2	4			
Total	53	100.0			
**Faculty member level**			**Students level**		
	n	%		n	%
Teaching assistant	14	26	Foundation year	32	13
Lecturer	6	11	First year	34	14%
Assistant professor	22	42	Second year	51	21
Associated professor	11	21	Third year	29	12
Total	53	100	Internship year	77	32
			Recent graduate	28	12
			Total	241	100

Table 2 presents a comprehensive overview of the feedback provided on various aspects of a higher educational institution, grouped as admissions, facilities, research, faculty staff roles, curriculum, outcomes, and internships.

**Table 2:** descriptive statistics of the entire questionnaire

Items	**Faculty members**			**Students**		
	N		mean	N		mean
	Valid	Missing		Valid	Missing	
Admission						
**The period of study in your college is longer than other colleges**	53	0	3.34	234	7	4.66
**There is a balance in the admission between deferent disciplines**	49	4	4.05	236	5	4.39
**There are typical entry requirements**	52	1	4.12	241	0	4.43
Facilities						
**Bachelor programs in HI encourage individuals to resolve community issues**	53	0	4.10	239	2	4.08
**Faculty members and students are involved in evaluating Bachelor programs in HI**	53	0	4.43	241	0	4.37
**Students collaborate with the academic department to develop syllabuses at the end of the year**	53	0	4.23	239	2	4.34
**Student concerns and complaints are taken seriously and resolved**	53	0	4.51	130	11	4.45
**The school gives their students the opportunity to choose their supervisors and the dissertation topics**	53	0	4.30	236	5	4.34
Research						
**Research assistants are available to support the students and provide required articles and resources**	53	0	4.54	238	3	4.21
**All required information and resources are available to support students in their research**	53	0	4.09	239	2	4.01
Faculty staff roles						
**Faculty staff encourage their students to discuss and think critically**	53	0	2.23	241	0	2.33
**Faculty staff always available for advice and guidance**	53	0	4.34	240	1	3.90
**Students receive recommendations and advice from the faculty staff to improve their research**	53	0	4.01	241	0	4.34
**The school regularly invites specialist faculty staff from outside the university to learn from their experiences**	53	0	4.20	239	2	4.25
**Faculty staff use the most recent journals and articles in their curricula**	53	0	4.43	236	5	4.55
**The faculty staff present courses scientifically to comply with postgraduate courses and their goals**	53	0	3.90	241	0	3.34
**The faculty staff emphasize the use multiple sources in their curricula**	51	2	4.28	237	4	4.56
**The faculty staff link their curricula with the reality of the society and culture**	53	0	4.32	240	1	4.56
**The number of the faculty staff is commensurate with number of postgraduate students according to the global standard**	53	0	4.34	241	0	4.24
**The faculty staff at the school has sufficient experience to deliver courses in accessible ways**	52	1	4.09	234	7	4.80
**Clear criteria are available to evaluate the faculty staff**	53	0	4.24	241	0	4.65
Curriculum						
**Learning methods rely on a critical thinking approach**	52	1	3.21	233	8	4.32
**Teaching methods include group discussions in classes**	53	0	3.95	233	8	4.23
**Students work in groups to complete group projects**	50	3	4.32	232	7	4.55
**Content of bachelor programs in HI consider the students’ research needs**	53	0	4.44	236	5	4.34
**Syllabuses help to improve students’ research skills**	53	0	4.59	233	8	4.23
**The content of bachelor programs in HI syllabuses is in line with students’ disciplines**	53	0	4.31	233	8	3.45
**Students benefited from the syllabus in writing their research project**	53	0	3.22	231	10	3.22
**Department provides courses that consider the program’s needs**	53	0	3.50	230	11	2.54
**Syllabuses help to improve students’ ability to think critically**	53	0	4.54	233	8	3.43
**Tests used vary between substantive and editorial approaches**	53	0	3.90	241	0	3.40
**Students are asked to prepare a scientific report on curricula topics**	53	0	4.33	241	0	4.29
Facilities						
**The university has access to most of the databases and data centres for research**	53	0	3.77	241	0	2.55
**Students benefit from analysis software provided by the university**	53	0	3.30	241	0	4.32
**All the research facilities include labs, and the libraries include reading rooms and a wide range of available books**	53	0	4.03	234	7	4.14
**The university provides printing and data analysis services**	53	0	3.90	225	16	4.54
**Students benefit from internet services and research centres that are provided by the university**	52	1	4.77	235	6	4.34
**The school covers all expense required to attend and participate in conferences**	53	0	4.23	237	4	4.55
**Students are able to access journals and databases from home**	53	0	4.73	241	0	4.69
**The most advanced technology is used to deliver the courses**	51	2	3.13	241	0	3.22
Outcome						
**Bachelor programs in HI meet student aspirations**	53	0	4.17	232	9	4.46
**Bachelor programs in HI contribute to achieving the community needs**	53	0	4.12	241	0	4.58
**Students graduate bachelor programs in HI with competencies**	53	0	4.21	241	0	4.43
**Internship**						
**Internship duration**	53	0	2.32	241	0	3.80
**Internship program improves the students’ skills and competencies**	53	0	4.88	246	5	4.45
**Internship program prepares students for the job market**	53	0	4.67	237	4	4.54
**Internship program covers all skills required to get an appropriate job in HI**	53	0	3.56	237	4	3.12
**Internship program duration is sufficient for undergraduate level**	53	0	2.23	241	0	4.92
**Internship program reflects all courses that are taught courses in the bachelor’s degree in HI**	53	0	4.81	233	8	4.85

Table 3 illustrates the correlation coefficients between different educational factors for the two groups.

**Table 3:** Correlations between eight variables

Variables		Facilities	Students’ Involvement	Curriculum	Research	Admission	Roles of Faculty Staff	Outcome	Internship
**Facilities**	Faculty	-							
	Students								
**Students’ Involvement**	Faculty	.81^b^	-						
	Students	.43^b^							
**Curriculum**	Faculty	.79^b^	.86^b^	-					
	Students	.38^b^	.73^b^						
**Research**	Faculty	.63^b^	.67^b^	.54^b^	-				
	Students	.46^b^	.60^b^	.55^b^					
**Admission**	Faculty	.09	.24	.16	.61^b^	-			
	Students	.55^b^	.40^b^	.34^b^	.55^b^				
**Roles of Faculty Staff**	Faculty	.49^b^	.65^b^	.68^b^	.61^b^	.59^b^	-		
	Students	.43^b^	.57^b^	.59^b^	.71^b^	.45^b^			
**Outcome**	Faculty	.43^b^	.53^b^	.56^b^	.41^a^	.27	.81^b^	-	
	Students	.33^b^	.64^b^	.69^b^	.48^**^	.42^b^	.71^b^		
**Internship**	Faculty	.21	.16	.14	.30	.13	.14	-.23	-
	Students	.14^a^	.33^b^	.37^b^	.34^b^	.12	.45^b^	.42^b^	

^a^Correlation is significant at the 0.05 level (2-tailed)

^b^Correlation is significant at the 0.01 level (2-tailed)

Table 4 presents a comparative analysis of the mean scores of various educational variables among students based on their current level of study. This interpretation focuses primarily on significance (sig.), which was provided for each variable to determine the statistically significant differences between groups.

**Table 4:** Comparisons between mean scores of variables among students based on current study level

		**Mean Square**	**F**	**Sig**
Facilities	Between Groups	.191	1.063	.343
	Within Groups	.163		
Students’ Involvement	Between Groups	.582	1.925	.096
	Within Groups	.307		
Curriculum	Between Groups	.987	4.374	.001
	Within Groups	.230		
Research	Between Groups	.513	.806	.612
	Within Groups	.705		
Admission	Between Groups	.634	1.137	.378
	Within Groups	.613		
Roles of Faculty Staff	Between Groups	.132	.389	.861
	Within Groups	.321		
Outcome	Between Groups	1.398	6.203	.000
	Within Groups	.231		
Internship	Between Groups	.209	1.211	.322
	Within Groups	.181		

^*^p < .05

Of the educational variables listed, two showed significant differences between student groups. Curriculum has a *p*-value of 0.001, indicating a highly significant difference between the groups. Similarly, Outcome stands out with a *p*-value of 0.000, suggesting an extremely significant variation among the study levels. 

Table 5 elucidates a comparative assessment of the mean scores for Admission, Roles of Faculty Staff, Outcome, and Curriculum between faculty members and students in relation to an internship program.

**Table 5:** Comparisons between mean scores in terms of internship program

		Mean Square	F	Sig
Admission	Faculty	.854	.950	.448
		.899		
	Student	1.19	2.02	.094
		.589		
Roles of Faculty Staff	Faculty	.080	.235	.916
		.342		
	Student	1.44	5.11	.001
		.283		
Outcome	Faculty	.213	.302	.874
		.704		
	Student	1.74	7.75	.000
		.225		
Curriculum	Faculty	.208	1.33	.280
		.156		
	Student	.864	5.23	.001
		.165		
Students’ Involvement	Faculty	.065	.085	.986
		.762		
	Student	1.63	2.40	.052
		.679		
Research	Faculty	.854	.950	.448
		.899		
	Student	1.19	2.02	.094
		.589		
Facilities	Faculty	.826	.919	.408
		.898		
	Student	.435	.720	.488
		.604		

^*^p < .05

Roles of Faculty Staff and Outcome for students exhibit significant differences with *p*-values of 0.001 and 0.000, respectively, suggesting notable variations in perceptions between the two groups regarding these aspects of the internship program. These values were well below the 0.05 threshold, underscoring their statistical relevance.

## Discussion

This study aimed to provide a comprehensive examination of accredited HI academic programs in Saudi Arabia. While the literature has rarely expounded on HI in Saudi Arabian universities, especially those accredited by the NCAAA, little is known about HI programs in Saudi Arabian universities, especially bachelor’s degrees.

The assessed criteria included several academic factors, such as student involvement, academic outcomes, and research, as well as logistical factors, including facilities, admission processes, personnel, and internships. Initially, students expressed a high level of satisfaction towards their academic programs. There were similarities between the attitudes expressed by students in this study and those described by Khan et al. [41]. This outcome is contrary to those of Rawas and Yasmeen [42] and Al-Natour [43], who found that students were less satisfied with their academic programs. The results of this study are mostly in accordance with the current literature on how HI has facilitated the electronic management of health information [1, 5], whereas the results indicating computational emphasis match the current literature [1], as HI programs offered in Saudi Arabian universities were found to have varying degrees of satisfaction among students. When looking at individual variables, the results of this study seem to be consistent with other studies that found a very high level of satisfaction among students towards research, the role of faculty members, and facilities [44].

A positive correlation was observed between college facilities and other academic and logistical characteristics. This finding suggests that students perceive an enhancement in their academic abilities when universities offer improved facilities. This study supports evidence from previous observations (e.g. [45]). Furthermore, a noteworthy association was observed among the eight factors as well as between each academic variable and each logistic variable. This suggests that the variables are interconnected and cannot be viewed in isolation. Additionally, this observation suggests that alterations in any of the factors could impact the remaining variables, potentially leading to cumulative effects that could be either advantageous or detrimental to the program. This implies that universities must not focus solely on academic brilliance or logistical aspects of their degree programs; instead, they must consider the integration of all factors to achieve a comprehensive level of student satisfaction. These findings match those observed in earlier studies [46, 47].

This study also aimed to compare the perceptions of faculty members and students. In general, both faculty members and students had high evaluations for many of the categories presented. For instance, both groups highly regarded the research support and facilities offered by the institution. However, noticeable differences are observed in certain areas. In the Admission category, students rated the statement ‘The period of study in your college is longer than other colleges’, much higher than faculty, indicating that students might feel the duration of their program is longer in comparison to similar programs. However, the faculty members showed more confidence in the balance of admissions between different disciplines.

Second, regarding the Roles of Faculty Staff and Facilities, students consistently gave higher ratings in most areas compared to faculty members. For example, students felt more strongly that the faculty emphasised the use of multiple sources in their curricula and that they linked the curriculum with the reality of society and culture. Interestingly, students also rated the faculty staff’s experience of delivering courses in a simple way higher than the faculty members did themselves. This suggests that students valued and recognised the teaching methods and efforts of the faculty staff.

Lastly, in the Internship category, there was a significant difference in perception regarding the internship duration. While faculty members found the duration to be much shorter (rating it at 2.32), students rated it much higher (3.80), suggesting that they felt it was relatively longer or more adequate. Moreover, both groups highly regarded the internship program's ability to reflect all taught courses in the bachelor’s degree in HI, with students rating it slightly higher. In addition, the results showed that first-year students had higher beliefs about curricula than third-year students, which might be because third-year students have started being introduced to the work field through internships and have realised that the curriculum is fully sufficient for real-world application. This could also explain why first-year students had higher expectations of outcomes than third-year students, as they believed that their undergraduate years would be sufficient to fully equip them for the work field. This finding is contrary to those of previous studies which suggested that internships show no significant differences among students [48, 49].

This study offers an in-depth understanding of HI programs in SA by drawing insights from two distinct populations. This introduces a valuable tool for assessing bachelor's degrees, particularly those in the health domain, using a central internship component. Moreover, it can serve as a beneficial guide for educational policymakers, program tutors, and curriculum developers. This study further highlights various indicators that pinpoint the strengths and shortcomings of HI programs. Moreover, a strength of this study is that it includes the perceptions of both students and faculty members. However, this study encountered some limitations, including the use of self-reported methods. While these methods may have been the only accessible tool for data collection, they constitute a potential threat to the internal validity of the study, as Heppner and Wampold [50] showed. With self-report methods, participants’ responses could be biased, or they may become ashamed and not provide accurate information. For instance, students might show a social desirability bias when asked about the effectiveness of an educational program, and they might exaggerate the benefits of the programs if they felt they were not able to comprehend some of the program courses. In some instances, students might also guess the study’s objectives and provide skewed information that could either confirm or challenge the researcher’s hypothesis.

Suggestions for future research include controlling the independent variables of the study through semi-structured one-on-one interviews that address variables such as student involvement and academic outcomes without ascribing any sense of liability or responsibility to students or staff, which could make it easier for them to provide their honest inputs. An inductive thematic analysis could be introduced in addition to this quantitative cross-sectional study as a mixed-methods research design would provide more insight into both the qualitative and quantitative aspects of the research question. Moreover, future research may need to compare HI programs that are and are not yet accredited by the NCAAA.

## Conclusions

This study offers a comprehensive analysis of HI programs accredited by the NCAAA at Saudi Arabian universities, illuminating the perceptions of both faculty members and students. Highlighting the significance of intertwined academic and logistic factors in shaping student satisfaction, this study emphasises the importance of considering both realms for holistic educational success. Notably, the perception disparity between students and faculty in areas such as Admission, Faculty Roles, and Internships sheds light on areas for improvement and alignment. The study also evaluated other factors, including Facilities, Curriculum, Research, Student Involvement, and Outcomes. While the results align with the current literature, the self-reported methodology employed poses inherent biases, potentially affecting the study's internal validity. Recommendations for future work emphasise the adoption of mixed methods, in-depth interviews, and comparisons between accredited and non-accredited HI programs to ensure a richer, multidimensional understanding of HI education in Saudi Arabia.

### Supplementary Information

**Additional file 1. **

## Data Availability

The datasets used and analysed in the current study are available from the corresponding author upon reasonable request.

## Abbreviations

HI: Health Informatics
IMIA: International Medical Informatics Association
MoE: Ministry of Education; SPSS, Statistical Package for Social Sciences
