# Supplementary material for: Perceptions of students and faculty on NCAAA-accredited health informatics programs in Saudi Arabia: an evaluative study
Source: BMC Med Educ. 2024 Mar 15;24:296. doi: 10.1186/s12909-024-05065-2 (PMC10943920; doi:10.1186/s12909-024-05065-2)
Supplement: Supplementary file 1 — Additional file 1. [file 12909_2024_5065_MOESM1_ESM.docx]

# Appendix 1

**Study questionnaire**

| Facilities | Scale |
| --- | --- |
| The university has an access to most of the data basis and data centres for research |  |
|  | Strongly disagree ( ), Disagree ( ), Neutral ( ), Agree ( ), Strongly agree ( ) |
| HI students benefited from analysis software that provided by the University | Strongly disagree ( ), Disagree ( ), Neutral ( ), Agree ( ), Strongly agree ( ) |
|  |  |
| All research facilities are available include labs, reading rooms at the library, and wide rang wide books at the library. | Strongly disagree ( ), Disagree ( ), Neutral ( ), Agree ( ), Strongly agree ( ) |
|  |  |
| The university provide printing and data analysis services. |  |
|  | Strongly disagree ( ), Disagree ( ), Neutral ( ), Agree ( ), Strongly agree ( ) |
| HI students benefited from the internet services and research centres that provided by the University. |  |
|  | Strongly disagree ( ), Disagree ( ), Neutral ( ), Agree ( ), Strongly agree ( ) |
| The school cover all expense required to attend and participation in conferences. |  |
|  | Strongly disagree ( ), Disagree ( ), Neutral ( ), Agree ( ), Strongly agree ( ) |
| HI students are able to access journals and data basis from home. | Strongly disagree ( ), Disagree ( ), Neutral ( ), Agree ( ), Strongly agree ( ) |
|  |  |
| The most advanced technology are used to deliver the courses. |  |
|  | Strongly disagree ( ), Disagree ( ), Neutral ( ), Agree ( ), Strongly agree ( ) |

| students’ involvements | Scale |
| --- | --- |
| Bachelor programs in HI encourage to figure the community issues | Strongly disagree ( ), Disagree ( ), Neutral ( ), Agree ( ), Strongly agree ( ) |
| Faculty members and students are involved in the evaluation of HI programs | Strongly disagree ( ), Disagree ( ), Neutral ( ), Agree ( ), Strongly agree ( ) |
| Students participate with the academic department to develop the syllabuses at the end of the year | Strongly disagree ( ), Disagree ( ), Neutral ( ), Agree ( ), Strongly agree ( ) |
| Students concern and Complaints are taken seriously and resolve | Strongly disagree ( ), Disagree ( ), Neutral ( ), Agree ( ), Strongly agree ( ) |
| The school give their students the opportunity to choose their supervisors and the dissertations topics. | Strongly disagree ( ), Disagree ( ), Neutral ( ), Agree ( ), Strongly agree ( ) |

| Curriculum | Scale |
| --- | --- |
| Learning methods relay on a critical thinking approached | Strongly disagree ( ), Disagree ( ), Neutral ( ), Agree ( ), Strongly agree ( ) |
| Group discussion is a part of the teaching methods in class | Strongly disagree ( ), Disagree ( ), Neutral ( ), Agree ( ), Strongly agree ( ) |
| Group work is available to produce a group project | Strongly disagree ( ), Disagree ( ), Neutral ( ), Agree ( ), Strongly agree ( ) |
| HI programs content consider the students research needs | Strongly disagree ( ), Disagree ( ), Neutral ( ), Agree ( ), Strongly agree ( ) |
| The HI syllabuses help to improve students research skills | Strongly disagree ( ), Disagree ( ), Neutral ( ), Agree ( ), Strongly agree ( ) |
| The content of HI syllabuses in line with students disciplines | Strongly disagree ( ), Disagree ( ), Neutral ( ), Agree ( ), Strongly agree ( ) |
| HI students benefited from the syllabus in writing the dissertation | Strongly disagree ( ), Disagree ( ), Neutral ( ), Agree ( ), Strongly agree ( ) |
| Department provide modules with a consideration of the program needs | Strongly disagree ( ), Disagree ( ), Neutral ( ), Agree ( ), Strongly agree ( ) |
| HI syllabuses help to improve students ability for critical thinking | Strongly disagree ( ), Disagree ( ), Neutral ( ), Agree ( ), Strongly agree ( ) |
| Tests used vary between substantive and editorial | Strongly disagree ( ), Disagree ( ), Neutral ( ), Agree ( ), Strongly agree ( ) |
| Students asked to prepare a scientific report on curricula topics | Strongly disagree ( ), Disagree ( ), Neutral ( ), Agree ( ), Strongly agree ( ) |

| Research | Scale |
| --- | --- |
| Research assistants are available to support the students to provide the required articles and resources | Strongly disagree ( ), Disagree ( ), Neutral ( ), Agree ( ), Strongly agree ( ) |
|  |  |
| All required information and resources are available to support students in their research. | Strongly disagree ( ), Disagree ( ), Neutral ( ), Agree ( ), Strongly agree ( ) |
|  |  |

| Admission | Scale |
| --- | --- |
| The period of study in your college is longer than other colleges? | Strongly disagree ( ), Disagree ( ), Neutral ( ), Agree ( ), Strongly agree ( ) |
|  |  |
| There is a balance in the admission between deferent disciplines? | Strongly disagree ( ), Disagree ( ), Neutral ( ), Agree ( ), Strongly agree ( ) |
|  |  |
| There is typical entry requirement? | Strongly disagree ( ), Disagree ( ), Neutral ( ), Agree ( ), Strongly agree ( ) |
|  |  |

| Roles of faculty staff | Scale |
| --- | --- |
| Faculty staff encourage their students for discussion and critical thinking? | Strongly disagree ( ), Disagree ( ), Neutral ( ), Agree ( ), Strongly agree ( ) |
|  |  |
| Faculty staff always available for advice and guidance. | Strongly disagree ( ), Disagree ( ), Neutral ( ), Agree ( ), Strongly agree ( ) |
|  |  |
| HI students received recommendation and advice from the faculty staff to improve their research. | Strongly disagree ( ), Disagree ( ), Neutral ( ), Agree ( ), Strongly agree ( ) |
|  |  |
| The school invite specialist faculty staff from outside the University to benefit from their experiences on regular basis. | Strongly disagree ( ), Disagree ( ), Neutral ( ), Agree ( ), Strongly agree ( ) |
|  |  |
| Faculty staff use the most recent journals and articles in their curricula. | Strongly disagree ( ), Disagree ( ), Neutral ( ), Agree ( ), Strongly agree ( ) |
|  |  |
| The faculty staff present the courses in scientific way which comply with HI courses and their goals. | Strongly disagree ( ), Disagree ( ), Neutral ( ), Agree ( ), Strongly agree ( ) |
|  |  |
| The faculty staff emphasise on the using multiple sources in their curricula. | Strongly disagree ( ), Disagree ( ), Neutral ( ), Agree ( ), Strongly agree ( ) |
|  |  |
| The faculty staff link the curricula with the reality of the society and culture. | Strongly disagree ( ), Disagree ( ), Neutral ( ), Agree ( ), Strongly agree ( ) |
|  |  |
| The number of the faculty staff is commensurate with number of HI students according to the global standard. | Strongly disagree ( ), Disagree ( ), Neutral ( ), Agree ( ), Strongly agree ( ) |
|  |  |
| The faculty staff at the school has sufficient experiences to deliver the courses in simple way. | Strongly disagree ( ), Disagree ( ), Neutral ( ), Agree ( ), Strongly agree ( ) |
|  |  |
| Clear criteria are available to evaluate the faculty staff. | Strongly disagree ( ), Disagree ( ), Neutral ( ), Agree ( ), Strongly agree ( ) |
|  |  |

| Outcome | Scale |
| --- | --- |
| HI programs meet the student aspirations? | Strongly disagree ( ), Disagree ( ), Neutral ( ), Agree ( ), Strongly agree ( ) |
|  |  |
| HI programs contribute to achieve the community needs? | Strongly disagree ( ), Disagree ( ), Neutral ( ), Agree ( ), Strongly agree ( ) |
|  |  |
| HI programs graduated competencies for HI market | Strongly disagree ( ), Disagree ( ), Neutral ( ), Agree ( ), Strongly agree ( ) |
|  |  |

| Internship | Scale |
| --- | --- |
| internship duration is appropriate for bachelor degree students | Strongly disagree ( ), Disagree ( ), Neutral ( ), Agree ( ), Strongly agree ( ) |
| internship program prepare students to the market | Strongly disagree ( ), Disagree ( ), Neutral ( ), Agree ( ), Strongly agree ( ) |
| internship program duration is sufficient for HI level | Strongly disagree ( ), Disagree ( ), Neutral ( ), Agree ( ), Strongly agree ( ) |
| internship program improves the students skills and competences | Strongly disagree ( ), Disagree ( ), Neutral ( ), Agree ( ), Strongly agree ( ) |
| internship program cover all skills required to get appropriate job in HI | Strongly disagree ( ), Disagree ( ), Neutral ( ), Agree ( ), Strongly agree ( ) |
| internship program reflect all thought courses in the bachelor degree in HI | Strongly disagree ( ), Disagree ( ), Neutral ( ), Agree ( ), Strongly agree ( ) |
